# Physiological Mouse Brain Aβ Levels Are Not Related to the Phosphorylation State of Threonine-668 of Alzheimer's APP

**DOI:** 10.1371/journal.pone.0000051

**Published:** 2006-12-20

**Authors:** Yoshitake Sano, Tadashi Nakaya, Steve Pedrini, Shizu Takeda, Kanae Iijima-Ando, Koichi Iijima, Paul M. Mathews, Shigeyoshi Itohara, Sam Gandy, Toshiharu Suzuki

**Affiliations:** 1 RIKEN, Brain Science Institute, Behavioral Genetics Wako, Japan; 2 Laboratory of Neuroscience, Graduate School of Pharmaceutical Sciences, Hokkaido University Sapporo, Japan; 3 Farber Institute for Neurosciences of Thomas Jefferson University Philadelphia, Pennsylvania, United States of America; 4 Cold Spring Harbor Laboratory, Cold Spring Harbor New York, United States of America; 5 Dementia Research Program, The Nathan S. Kline Institute for Psychiatric Research Orangeburg, New York, United States of America; University of Giessen, Germany

## Abstract

**Background:**

Amyloid-β peptide species ending at positions 40 and 42 (Aβ40, Αβ42) are generated by the proteolytic processing of the Alzheimer's amyloid precursor protein (APP). Aβ peptides accumulate in the brain early in the course of Alzheimer's disease (AD), especially Aβ42. The cytoplasmic domain of APP regulates intracellular trafficking and metabolism of APP and its carboxyl-terminal fragments (CTFα, CTFβ). The role of protein phosphorylation in general, and that of the phosphorylation state of APP at threonine-668 (Thr^668^) in particular, has been investigated in detail by several laboratories (including our own). Some investigators have recently proposed that the phosphorylation state of Thr^668^ plays a pivotal role in governing brain Aβ levels, prompting the current study.

**Methodology:**

In order to evaluate whether the phosphorylation state of Thr^668^ controlled brain Aβ levels, we studied the levels and subcellular distributions of holoAPP, sAPPα, sAPPβ, CTFα, CTFβ, Aβ40 and Aβ42 in brains from “knock-in” mice in which a non-phosphorylatable alanyl residue had been substituted at position 668, replacing the threonyl residue present in the wild-type protein.

**Conclusions:**

The levels and subcellular distributions of holoAPP, sAPPα, sAPPβ, CTFα, CTFβ, Aβ40 and Aβ42 in the brains of Thr^668^Ala mutant mice were identical to those observed in wild-type mice. These results indicate that, despite speculation to the contrary, the phosphorylation state of APP at Thr^668^ does not play an obvious role in governing the physiological levels of brain Aβ40 or Αβ42 *in vivo*.

## Introduction

Alzheimer's disease (AD) is characterized by abnormalities in post-translational processing of two proteins, the amyloid precursor protein (APP) and the microtubule associated protein tau [1]. A “unifying hypothesis for Alzheimer's disease” has been suggested, according to which the aberration of a single post-translational modification, such as protein phosphorylation, might underlie both pathologies [2]–[4]. Both proteins are phosphoproteins [5]–[7], and cyclin-dependent protein kinase 5 (cdk5) and glycogen synthase kinase 3β (GSK-3β) are examples of protein kinases that can potentially phosphorylate both APP and tau [6]–[8].

A recent review summarizes the extensive literature linking protein phosphorylation and APP metabolism [9]. First messengers, such as neurotransmitters and hormones, impinge upon neurons and direct APP toward the cell surface and away from the *trans* Golgi network (TGN) and endocytic pathway, and hence away from the Aβ-generating β-/γ-secretase pathway [*ibid*]. At the cell surface, APP can be processed by a nonamyloidogenic pathway, known as the α-secretase pathway, via a process known as “regulated APP ectodomain shedding” [*ibid*]. With regard to Aβ generation, the regulated shedding phenomenon is noteworthy because hyperactivation of the α-secretase pathway can lead to relatively greater cleavage of APP by α-secretase(s), thereby reducing or completely abolishing Aβ generation [*ibid*]. Despite the well-documented observation that the same protein kinase (i.e., protein kinase C) controls both regulated shedding by α-secretase ***and*** the phosphorylation state of the APP cytoplasmic tail at serine-655 (Ser^655^), the phosphorylation state of APP at Ser^655^ is not involved in controlling regulated shedding of the APP ectodomain. Primary citations for this summary are included in [9].

The exclusion of an important role for the APP phosphorylation state in controlling regulated APP ectodomain shedding process raised an obvious question: “What role ***is*** played by physiological regulation of the direct phosphorylation of APP?”. First of all, in brain, APP phosphorylation predominantly involves Thr^668^, not Ser^655^ [6]–[8]. Phosphorylation at Thr^668^ is detectable in mature APP (mAPP) but not immature APP (imAPP) [7], suggesting that the phosphorylation state of Thr^668^ may regulate some aspect of APP maturation in the early secretory pathway. In addition to cdk5 and GSK-3β mentioned above, phosphorylation at Thr^668^ can be regulated by c-jun N-terminal kinases (JNK) [10], [11]. With regard to the physiological role of APP phosphorylation, the phosphorylation state of Thr^668^ has been reported to modulate outgrowth of neurites in PC12 cells [12], [13], the tethering to APP of the adaptor proteins FE65 [14], [15] and X11-L [16], and the interaction of APP intracellular domain fragment (AICD) with FE65 [14], [15]. Based on these data, we have proposed a model wherein the phosphorylation state of APP at Thr^668^ may govern the state of activation of an intracellular signaling cascade across APP that leads to generation and translocation of AICD, analogous to the well-characterized signaling cascade across Notch that leads to generation and translocation of the Notch intracellular domain [15].

Recently, the phosphorylation state of APP Thr^668^ has been proposed to control interaction of the APP cytoplasmic tail with the prolyl isomerase Pin1 [2], [3], [17]. Interaction of Pin1 with APP Thr^668^ has been predicted to have important effects on generation of the Aβ peptide that not only accumulates in amyloid plaques but also forms oligomers that have recently been proposed to be the proximate mediators of neurotoxicity [18]. One report indicated that Aβ levels were decreased in the brains of Pin1-deficient mice [17], while another report described the opposite effect, i.e., that Pin1 deficiency increased Aβ generation [2]. In work unrelated to Pin1, Tsai *et al* proposed a model wherein the phosphorylation state of APP Thr^668^ would be pivotal in modulating the subcellular distribution of APP and generation of Aβ [4] and/or the amyloidogenic γ-secretase cleavage of APP CTFs [19]. The potential importance of APP Thr^668^ phosphorylation was further emphasized in a recent review [3]. Because all these reports and reviews hinged on attribution of some biological significance to the phosphorylation state of APP Thr^668^, we investigated *in vivo* brain APP metabolism and Aβ levels in mutant mice generated by knocking into their genome an APP gene containing a non-phosphorylatable alanyl substitution at position 668. Here we report characterization of these mice, including the quantification and subcellular distribution of all standard APP metabolites.

## Results

### Generation of threonine-668 phosphorylation-site mutant mice

**Figure 1:**
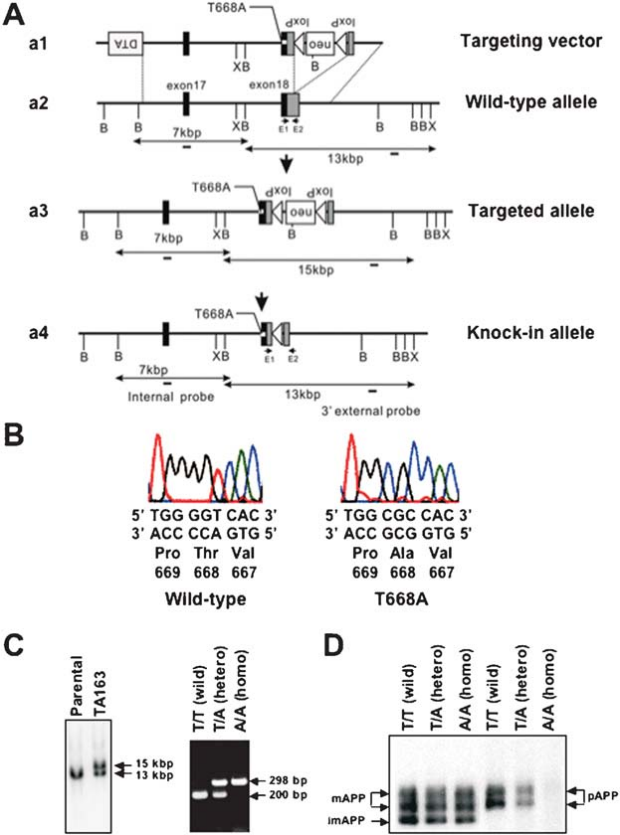
Generation of Thr^668^Ala Knock-in Mutant Mice. (A) The targeting vector (a1), a partial map of the APP gene (a2), the resultant targeted allele (a3), and the knock-in allele after Cre recombination (a4) are illustrated. Filled boxes denote coding sequences of exons 17 and 18. Shaded parts in exon18 correspond to the 3′ non-coding region. A substitution is represented by a dot in exon 18. B, *BamHI*; X, *XhoI*. Probes for Southern blot analysis for screening of targeted ES clones are indicated with small bars in (a2–4). PCR primers (E1 and E2) for genotyping of mice are indicated with small arrows in (a2 and a4). (B) Verified sequences from wild-type (T/T), and Thr^668^Ala mutation homozygotes (A/A). The Thr^668^ flanking genomic region was amplified using mouse tail DNA as the template by PCR and sequenced. (C) Southern blot analysis for targeted ES cells and PCR analysis for genotyping wild-type and knock-in mouse lines. DNA from G418-selected ES cells was digested with *XhoI* and analyzed by Southern blotting with a 3′ external probe. The 13-kb and 15-kb fragments represent wild-type and targeted alleles, respectively. PCR fragments of 200-bp and 298-bp represent wild-type and knock-in alleles, respectively. (D) Immunoblot analysis of mouse whole brain. APP was immunoprecipitated with anti-pan APP polyclonal antibody followed by immunoblotting with anti-pan APP antibody UT-421 or anti-phospho-Thr^668^-specific antibody UT-33 [7]. APP from homozygotes has no immunoreactivity with UT-33 although the APP expression levels detected by UT-421 were indistinguishable from those of wild-type mice. Mature APP (mAPP; *N*- and *O*-glycosylated form), immature APP (imAPP; *N*-glycosylated form), and phosphorylated APP (pAPP) are indicated with arrows.

We constructed targeting vectors in which an alanine (A) was substituted for threonine (T) at position 668 in exon 18 (Fig. 1A). Targeted C57BL/6-derived MS12 embryonic stem (ES) cells were injected into Balb/c blastocysts. Chimeras were bred to C57BL/6 mice to generate heterozygotes. Fertilized eggs from the heterozygotes were then injected with a Cre-expression plasmid vector to delete the Pgk-neo gene cassette from the germ line via the Cre-loxP system [20], and resulting progeny carrying a substituted allele without the Pgk-neo gene cassette were backcrossed to C57BL/6 mice for at least 7 generations, and then intercrossing was performed to produce Thr^668^Ala homozygous mice. Thus, the mutant lines used in this study were coisogenic to the C57BL/6 strain. Seven generations of crossing is considered acceptable for mice generated by a coisogenic strategy [21]. The success of the mutagenesis was confirmed by sequencing (Fig. 1B), Southern blot, and polymerase chain reaction (PCR) analysis (Fig. 1C). We also confirmed the mutation by immunoblot analysis using anti-phospho-APP Thr^668^ specific antibody (Fig. 1D). There was no anti-phospho-APP Thr^668^ antibody staining in homozygotes (A/A), while heterozygotes (T/A) had approximately half the level of the staining that was observed in wild-type (T/T). Anti-pan APP-specific antibody staining revealed unaltered expression levels of APP in all genotypes (Fig. 1D).

### Thr^668^Ala mutation did not affect brain development or structure

**Figure 2:**
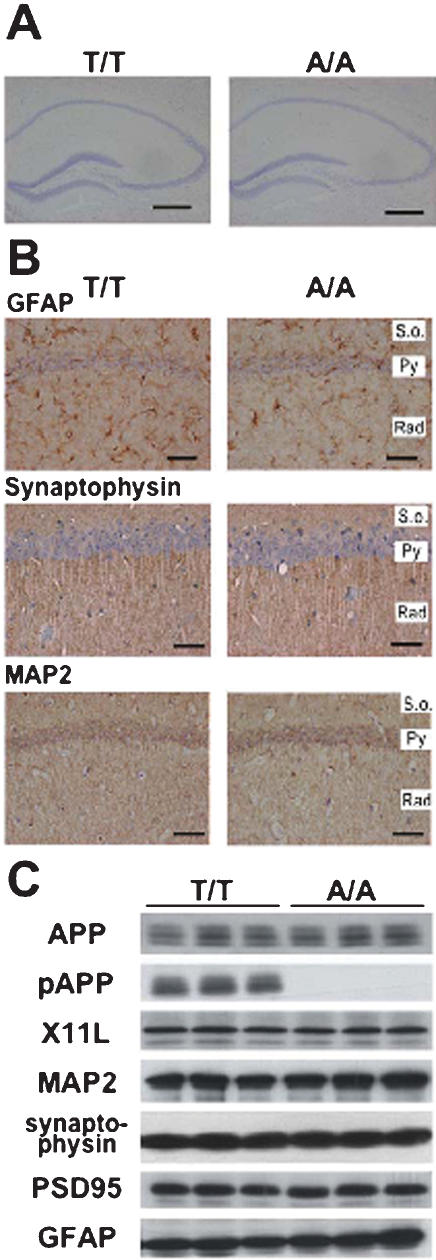
Normal brain structure and expression and distribution of proteins related to neuron and glia in Thr^668^Ala mutant mice. (A) Nissl-stained hippocampal sections show no difference between wild-type (T/T) and Thr^668^Ala mutation homozygotes (A/A). Scale bars represent 500 µm. (B) Immunohistochemical analysis in CA1 hippocampal region of aged (>12 mo-old) mice. Immunostaining for GFAP (astroglial marker), synaptophysin (presynaptic marker), and MAP-2 (neuronal dendritic marker) in hippocampal CA1 region of wild-type (T/T) and Thr^668^Ala mutant (A/A) mice are shown. S.o., stratum oriens; Py, pyramidal cell; Rad, stratum radiatum. Scale bars represent 50 µm. (C) Western blot analysis of APP, X11L, MAP2, synaptophysin, PSD95 (postsynaptic marker), and GFAP from the brains of 12 mo-old wild-type (T/T) and Thr^668^Ala mutant (A/A) mice.

The A/A mice had no gross cytoarchitectural abnormalities as revealed in Nissl-stained sections of the hippocampal region from A/A mice (Fig. 2A). Other brain regions were also histologically normal (data not shown). Furthermore, immunohistochemical and/or immunoblotting analyses for synaptophysin (presynaptic marker), microtubule-associated protein (MAP2; dendritic marker), glial fibrillary acidic protein (GFAP; astroglial marker), X11L (APP binding protein), and PSD95 (postsynaptic marker) revealed no abnormalities in A/A mice, even at ages of 12 mo or older (Fig. 2B and C). These results contrast to those from APP-null mutant mice that showed marked decreases of synaptophysin, synapsin, and MAP-2 [22], a reduction in dendrite length [22], and gliosis [23]. The normal phenotype of APP Thr^668^Ala mice suggests that this mutation does not cause a major loss-of-function of APP.

### Analysis of APP and its metabolites in APP Thr^668^Ala knock-in mice

**Figure 3:**
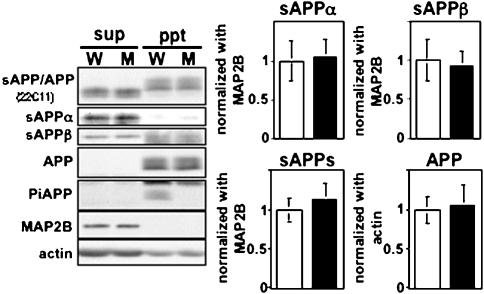
APP and sAPP in wild-type and Thr^668^Ala mutant mouse brain. (Left) APP and sAPP in wild-type (W) and mutant (M) mouse brain. Homogenates of brains taken from 12-month-old mice were fractionated as described and analyzed by immunoblotting for sAPP and APP holoprotein (22C11), APP (APP/C), phosphorylated APP (pThr^668^APP), sAPPα, sAPPβ and MAP2B. Protein bands observed in the insoluble fraction (ppt panel) of sAPPβ are non-specific. (Right) sAPP and APP levels are displayed. The densities of the bands from soluble APP (sAPP) were standardized to the densities of MAP2B, and those from APP were standardized to the densities of actin. All were normalized to unity for wild-type mice (1.0). The bars indicate means±S.D. (N.S.; n = 4).

**Figure 4:**
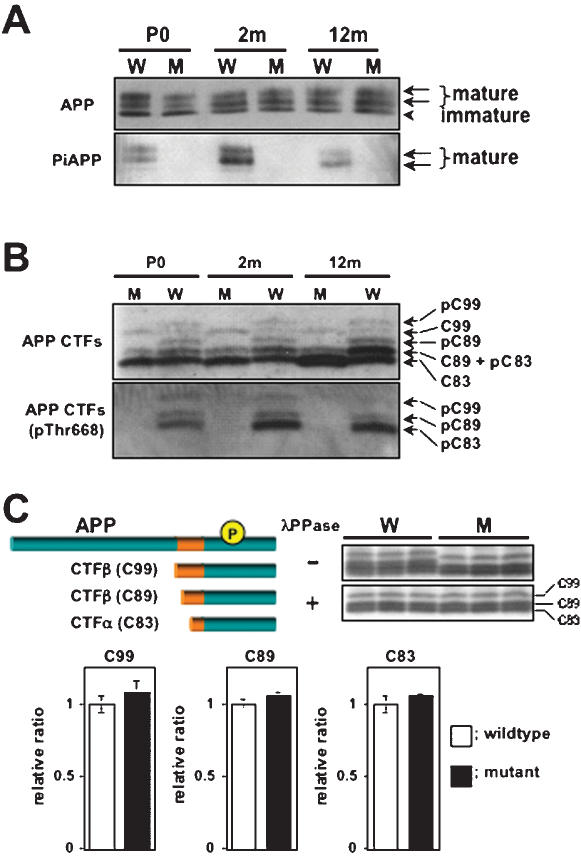
Aging-dependent phosphorylation of APP and APP CTFs and quantification of CTFs in wild-type and Thr^668^Ala mutant mice brain. (A) APP phosphorylation state in brains of post-natal day 0 (P0), young adult (2-month), and aged adult (12-month) mice. The upper panel was probed with anti-pan-APP C-terminal antibody G369, and the lower panel was probed with an anti-phospho-threonine-^668^-specific antibody. W, wild-type mouse; M, Thr^668^Ala mutant mouse. (B) APP carboxyl-terminal fragments (APP CTFs) in wild-type (W) and mutant (M) mouse brain. C99 and C89 are products resulting from cleavage of APP by BACE, while C83 results from cleavage of APP by ADAM-10/-17. PhosphoC99 (pC99), phosphoC89 (pC89), and phosphoC83 (pC83) are all mono-phosphorylated at Thr^668^, and these peptides are numbered here according to standard APP_695_ nomenclature. (C) Expression levels of CTFα and CTFβ in middle aged wild-type and Thr^668^A mutant mouse brain. Various species of CTF are schematically represented at the left and indicated at right with bars. Samples were electrophoresed after treatment with either buffer or λ phosphatase (λ PPase). The amounts were normalized to unity for wild-type mice (1.0). The bars indicate means±S.D. (N.S.; n = 6).

The brains of APP Thr^668^Ala knock-in mice were analyzed for expression of APP and for steady-state levels of its metabolites (Figs. 3 and 4; Table 1). APP Thr^668^ phospho-state specific antibodies were used to establish that the knocked-in protein was not phosphorylated at Thr^668^ (Fig. 3 and Fig. 4A). Amino-terminal fragments of APP cleaved by α- (sAPPα) and β- (sAPPβ) secretases were quantified and compared with those of wild-type mouse brain (Fig. 3). No differences of total amount of sAPP, sAPPα, and sAPPβ were detected between Thr^668^Ala knock-in and wild-type mice.

**Table 1:**
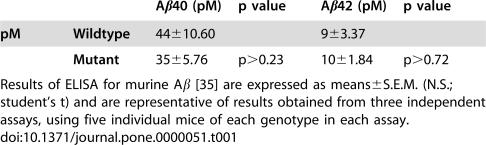
Table 1. Aβ40 and Aβ42 levels in brains from 24-month-old wild-type and Thr^668^Ala mutant mice.

		Aβ40 (pM)	p value	Aβ42 (pM)	p value
**pM**	**Wildtype**	44±10.60		9±3.37	
	**Mutant**	35±5.76	p>0.23	10±1.84	p>0.72

Results of ELISA for murine Aβ [35] are expressed as means±S.E.M. (N.S.; student's t) and are representative of results obtained from three independent assays, using five individual mice of each genotype in each assay.

Next, we examined protein levels of APP C-terminal fragments (CTFs) by immunoblot analysis using anti-APP cytoplasmic tail antibodies (Fig. 4). The phosphorylation levels of APP and CTFs are relatively lower at the birth (P0), they increase during growth (2-mo), and they slightly decrease during aging (12-mo). Cleavage by α-secretase generates CTFα, which is composed of the carboxyl-terminal 83 amino acids of APP and is also known as C83. β-secretase can cleave APP at either the peptide bond N-terminal to D^1^ (preferred) or at the bond N-terminal to E^11^; hence, CTFβ and CTFβ′ fragments, C99 and C89 [24], respectively, are generated, with the preponderant species being CTFβ′/C89 [24]. Because these CTFs are phosphorylated at Thr^668^ (Fig. 4B) and generate complicated patterns [25], [26], we treated samples with lambda protein phosphatase (λPPase) in order to identify and quantify phospho- and dephospho-CTF species precisely (Fig. 4C). Treatment of immunoprecipitates containing these CTFs with λPPase resulted in the appearance of three discrete bands corresponding to dephospho-forms of C99, C89, and C83, all of which were present at identical levels in both wild-type and Thr^668^Ala knock-in mice.

Endogenous murine Aβ40 and Aβ42 levels were also detected in brain. Rodent Aβ is much less prone to aggregation than is human Aβ [27], and therefore little, if any, insoluble Aβ is typically detected in the wild-type mouse brain. Nevertheless, we extracted mouse brain Aβ with 6 M guanidine chloride/TBS (the standard protocol for dissolving insoluble Aβ), and we quantified the solubilized Aβ. The levels of Aβ40 and Aβ42 in the brains of wild-type and Thr^668^Ala knock-in mice were indistinguishable (Table 1).

**Figure 5:**
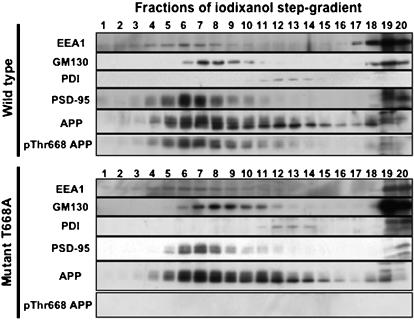
Hemi-brains from wild-type (upper panel of six blots, labeled “Wild-type”, far left) or mutant (lower panel of six blots; labeled “Mutant T^668^A”, far left) 5-month old mice were used for fractionation on iodixanol density-gradients as described (4). Equal aliquots (according to volume) were analyzed by immunoblotting with antibodies as specified: anti-early endosome antigen 1 (Transduction laboratories, EEA1, top panel), anti-*cis*-Golgi matrix protein (Transduction laboratories, GM130, second panel), anti-protein disulfide isomerase (Stressgen, PDI, a marker for the endoplasmic reticulum, third panel), anti-post synaptic density 95 (Transduction laboratories, PSD95, a synaptic membrane marker, fourth panel), anti-pan-APP (G369, fifth panel) and anti-phospho-threonine^668^ APP (Cell Signaling, bottom panel).

We fractionated mouse brains using an iodixanol gradient fractionation system identical to that described in the published cell culture study [4] (Fig. 5). Using homogenates of wild-type mouse brains, we observed that total mAPP as well as the corresponding phosphorylated form were largely recovered in fractions 5–9, where GM130 (Golgi marker protein, concentrated in fractions 7–9) and EEA1 (early endosomal protein, concentrated in fractions 4–8) were co-distributed (upper panel). Total Thr^668^Ala mAPP in knock-in mutant mouse brains was identical in levels and distribution except that, as expected, there was no detectable phospho-mAPP (Fig. 5). These data do not support the prediction [4] that the phosphorylation state of APP at Thr^668^ modulates its subcellular distribution in brain *in vivo*.

## Discussion

Generation, aggregation, and deposition of Aβ are key steps in the pathogenesis of AD [1]. Aβ is generated in the process of intracellular trafficking of APP, which is type I membrane protein. Following biosynthesis of APP, APP enters the endoplasmic reticulum where the protein is subjected to *N*-glycosylation (imAPP) and then transported to Golgi and subjected to *O*-glycosylation (mAPP). In neurons, membrane vesicles containing mAPP may be transported to nerve terminals along the axon using a kinesin-dependent motor system or else may be transcytosed and localized in dendrites [25]. At least some APP is exposed on the plasma membrane. This cell-surface APP, as well as APP in the *trans* Golgi network, is conveyed to the endocytic system via clathrin-coated vesicles [28]. Thus, APP might possibly be cleaved in one or several of compartments; i.e., the constitutive secretory pathway, the plasma membrane, and/or the endocytic system.

The short cytoplasmic domain of APP plays an important role in the regulation of intracellular APP trafficking and contains several functional motifs such as 681-GYENPTY-687, where several regulatory proteins interact [25]. One motif in the cytoplasmic domain of APP that has been proposed to be functionally important is 667-VTPEER-672 which forms a type I β-turn and amino-terminal helix capping box structure and contributes to the stability of a carboxyl-terminal helix structure [29], [30]. The phosphorylation of Thr^668^, situated within this motif, induces significant conformational change in the cytoplasmic domain, changing its interaction with FE65 [14], [15]. Because of these structural phenomena, phosphorylation of APP at Thr^668^ has been proposed by various groups to control either the metabolism of APP, some APP-related physiological function(s), and/or some FE65-mediated events.

Some biological phenomena appear to correlate with APP phosphorylation. Neurite outgrowth in PC12 cells has been reported to correlate with the phosphorylation of APP, and a Thr^668^Glu substitution remarkably reduced neurite extension response following treatment with NGF [12]. Membrane proteins, including APP, act to tether FE65 to Golgi membranes, and phosphorylation of APP releases FE65 from its membrane-protein anchor [15], [31]. The released FE65 may translocate into the nucleus and activate gene expression [32].

However, the role for the phosphorylation state of APP at Thr^668^ in the regulation of APP metabolism in brain *in vivo* has not been clarified. As mentioned above, Tsai and colleagues reported that amino acid substitutions at Thr^668^ alter the intracellular distribution of APP in cultured cells [4]. Alternatively, a recent model from Lu and colleagues holds that the prolyl isomerase Pin1 interacts with the phosphorylated form of APP at Thr^668^ , and that the interaction of Pin1 with APP Thr^668^ has important effects on Aβ levels [2; for review, see 3]. Aβ levels in Pin1-deficient brains have been studied not only by Lu and colleagues [2], but they have also been studied independently by Akiyama and colleagues [17]. These groups reported directly contradictory results, however, with one group reporting that Aβ levels were *decreased* in the brains of Pin1-deficient mice, while the other reported that Aβ levels were *increased* in the brains of Pin 1-deficient mice [2], [17]. Thus, since four recent and highly visible papers [2, 4,17; rev in 3] have emphasized the potential importance of APP Thr^668^ phosphorylation state in controlling brain Aβ levels, we investigated brain APP metabolism and Aβ levels in mutant mice generated by knocking into the mouse genome an APP gene containing a non-phosphorylatable alanine substitution at position 668 of APP.

The levels and subcellular distribution of APP and all its metabolites (sAPPα, sAPPβ, CTFα, CTFβ, Aβ40, Aβ42) from the brains of wild-type and APP Thr^668^Ala mutant mice were indistinguishable. Hence, contrary to widely publicized models [2, 4, 17, rev in 3], the phosphorylation state of APP Thr^668^ does not play an obvious role in governing physiological levels of brain Aβ *in vivo*.

Of note, during the final stages of production of this manuscript, a report appeared from Cruz *et al* [36] in which a revised model is presented wherein changes in APP metabolism caused by cdk5 are attributed to increased levels of β-APP-site cleaving enzyme (BACE) in contrast to this group's previous proposal [4] that the state of phosphorylation of Thr^668^ was the most important modulator of Aβ levels and subcellular distribution of APP and its derivatives. This revised model of Tsai and colleagues, focusing on BACE levels rather than APP phospho-Thr^668^ levels [36], is entirely consistent with what we have reported here.

It is worth noting that we cannot, of course, rule out the possibility that pathological changes in APP Thr^668^ phosphorylation state might modulate its function or metabolism. Presently, we favor a model in which APP phosphorylation at Thr^668^ may regulate either some important physiological function of APP that is not directly linked to the proteolytic processing pathways that control Aβ levels in brain neurons *in vivo*. Evidence already exists that such functions might well include modulation of neurite outgrowth [12], [13] and/or intracellular signaling via AICD or FE65 [14], [15].

## Materials and Methods

### Production of mutant mice

Mutant mice were generated by a standard gene knock-in method using MS12 ES cell line derived from mice of the C57BL/6 background [33]. Briefly, a 13-kb genomic fragment from C57BL/6 mice containing exons 17 and 18 was used for constructing targeting vectors. The T^668^A point mutation (see Figure 1B) was introduced by PCR mutagenesis. For positive selection, the Pgk-neo gene cassette flanked by loxP sites was inserted at the EcoR1 site in the 3′ non-coding region of APP-exon 18. For negative selection, the diphtheria toxin A-fragment gene cassette derived from pMC1DT-A was added to the 5′ end of the targeting vector. After transfection of MS12 ES cells derived from the C57BL/6 mouse strain and selection with G418, targeted clones were identified by Southern blot analyses. Chimeras, generated by injection of the targeted MS12 ES cells into Balb/c blastocysts, were mated with C57BL/6 mice to obtain mutant heterozygotes. The Pgk-neo cassette flanked by loxP sites was excised by injecting the Cre recombinase expression vector, pCAGGS-Cre [20] into the pronucleus of heterozygous fertilized eggs. The mouse genotypes were determined by PCR, using tail DNA as the template and the primers (E1: 5′-CACATTGATTTCTTTGTGCCTG-3′ and E2; 5′-TCTGTACAATCATCCTGCAG-3′), which anneal to the *loxP* flanking regions. Fragments of 200 bp and 298 bp were amplified from wild-type and knock-in mutant alleles, respectively.

### Immunoblot analyses

Brains were homogenized in radioimmunoprecipitation (RIPA) lysis buffer containing 1 µM microcystin-LR, 5 µg/ml chymostatin, and 5 µg/ml leupeptin, and the clear supernatants were used for immunoblot analysis. Aliquots of the lysates (100 µg protein) were separated on SDS-PAGE (6% (w/v) polyacrylamide for APP, X11L, MAP2 and PSD95, or 12.5% (w/v) polyacrylamide for synaptophysin and GFAP, and analyzed by immunoblotting with anti-APP cytosolic domain-specific polyclonal antibody (pAb) G369, anti-phospho Thr^668^ (pAPP)-specific pAb (Cell Signaling Technology, MA), anti-X11L cytoplasmic domain (735-AMFRLLTGQETPLYI-749 of human X11L)–specific monoclonal antibody (mAb), anti-MAP2 mAb HM-2 (Sigma, St. Louis, MO), anti-synaptophysin mAb SVP-38 (Sigma), anti-PSD95 mAb Clone16 (Transduction Laboratories), anti-actin MAB1501 (Chemicon International Inc., CA) and anti-GFAP mAb GF12.24 (Progen, Germany), respectively. Immunoreactive proteins were visualized with an enhanced chemiluminescence system (ECL; Amersham Pharmacia Biotech, Uppsala, Sweden).

APP CTFs were detected by immunoblotting as described [34], with modifications as described. Equal aliquots of homogenates (100 µg) were separated by electrophoresis in a Tris-tricine gel (16% [w/v] polyacrylamide). The separated proteins were transferred to nitrocellulose membranes, the membranes were boiled in PBS for 5 min, and probed with anti-APP cytoplasmic domain antibody (Sigma). Immunoreactive proteins were again visualized with an ECL detection system (Amersham Pharmacia Biotech). For sAPP detection, brains of mice of various ages (14∼15 month-old and 24 month-old) were homogenized in TBS buffer (50 mM Tris-HCl, 150 mM NaCl containing 1 µM microcystin-LR, 5 µg/mL chymostatin, and 5 µg/mL leupeptin) and centrifuged at 200,000× *g* for 20 min at 4°C. The pellets were subjected to two additional cycles of resuspension in equal volumes of TBS and centrifugation for 5 min at 200,000× *g* at 4°C. Samples were then lysed in an equal volume of 2× RIPA buffer and sonicated. Protein concentrations were quantified using a BCA protein assay kit (Pierce). Aliquots containing 30 µg protein were separated in 6% (w/v) Tris-glycine SDS-polyacrylamide gels, transferred to nitrocellulose, probed with anti-sAPPβ (kindly provided by T. C. Saido), or anti-sAPPα antibody 2B3 (IBL, Takasaki, Japan) and immunoreactive protein species were detected using an ECL detection system.

### Immunocytochemistry

Aged mice (12 mo-old or older) were deeply anesthetized with Avertin and transcardially perfused with physiologic saline and then 4% (w/v) paraformaldehyde in 0.1 M Na-phosphate buffer pH7.4 at 4°C for 20 min. The brain was excised and post-fixed with the same fixative at 4°C overnight and cryoprotected in 30% (w/v) sucrose/phosphate-buffered saline (PBS). The brains were embedded into OCT compound (Sakura Fine Technical Co. Ltd., Tokyo, Japan) and frozen coronal sections (30 µm) were prepared. Immunohistochemical staining was performed using the ABC method (Vector Laboratories, Burlingame, CA). Sections were incubated with 0.1% (v/v) Triton X-100 in PBS for 30 min at room temperature followed by incubation with 0.3% (v/v) hydrogen peroxide in PBS for 30 min at room temperature to quench endogenous peroxidase activity. The sections were then blocked with 5% (v/v) horse serum in PBS at 4°C overnight. After an overnight incubation at 4°C with anti-GFAP (clone GF12.24), anti-synaptophysin (DAKO Corp., CA; clone SY38), or anti-MAP2 (Chemicon International, Inc., CA; clone MAB378) mAbs, the sections were further incubated with horse anti-mouse IgG conjugated to biotin (Vector Laboratories) for 1 h at room temperature, followed by the ABC complex. The peroxidase activity was revealed using diaminobenzidine as the chromogen.

### Quantification of Aβ

Endogenous mouse brain Aβ40 and Aβ42 were quantified with the ELISA system developed by Mathews and colleagues as described [35].
